# Use of External Fixator Device for Mandible Fracture Related to War Injury: A Systematic Review

**DOI:** 10.3390/jcm14093061

**Published:** 2025-04-29

**Authors:** Franck Masumbuko, Gregory Reychler, Olivier Cornu, Caroline Huart, Jean Cyr Yombi, Raphael Olszewski

**Affiliations:** 1 Unit of Maxillofacial and Reconstructive Surgery, Surgery Department, Hôpital Provincial Général de Référence de Bukavu, Université Catholique de Bukavu, Bukavu 285, Democratic Republic of the Congo; 2 Oral and Maxillofacial Surgery Lab (OMFS Lab), Neuro Musculo Skeletal Lab (NMSK), Institut de Recherche Expérimentale et Clinique (IREC), Université Catholique de Louvain (UCLouvain), 1200 Brussels, Belgium; raphael.olszewski@saintluc.uclouvain.be; 3 Service de Kinésithérapie et Ergothérapie, Institut de Recherche Expérimentale et Clinique (IREC), Cliniques Universitaires Saint Luc, Université Catholique de Louvain (UCLouvain), 1200 Brussels, Belgium; gregory.reychler@saintluc.uclouvain.be; 4 Division of Orthopedics and Musculoskeletal Trauma, Cliniques Universitaires Saint Luc, Université Catholique de Louvain (UCLouvain), 1200 Brussels, Belgium; olivier.cornu@saintluc.uclouvain.be; 5 Division of Ear, Nose and Throat (ENT), Cliniques Universitaires Saint Luc, Université Catholique de Louvain (UCLouvain), 1200 Brussels, Belgium; caroline.huart@saintluc.uclouvain.be; 6 Division of Internal Medicine and Infectious diseases, Cliniques Universitaires Saint Luc, Université Catholique de Louvain (UCLouvain), 1200 Brussels, Belgium; jean.yombi@saintluc.uclouvain.be; 7 Department of Oral and Maxillofacial Surgery, Cliniques Universitaires Saint Luc, Université Catholique de Louvain, 1200 Brussels, Belgium; 8 Department of Perioperative Dentistry, L. Rydygiera Collegium Medicum, Nicolaus Copernicus University, 85-067 Bydgoszcz, Poland

**Keywords:** external fixator, war injuries, mandibular fracture

## Abstract

**Background/Objectives**: In maxillo-facial high-velocity complex war injuries, a rigid internal fixation is inappropriate, and external fixation is suitable with described benefits. This systematic review aimed to summarize the literature regarding the benefits, side effects and complications of external fixators in the management of mandibular war-related injuries. **Methods**: An electronic search was performed in the databases of PubMed and Google Scholar in December 2024. The title and abstracts from retrieved items were read by two reviewers to identify studies within the selection criteria. Included articles had to be published in English up to December 2024 and related to external fixators used in mandibular fracture war injuries. **Results**: The search strategy initially identified 445 studies through PubMed and 987 studies through Google Scholar. Following the application of inclusion criteria, 12 articles were selected for this review, describing the use of an external fixator for a mandibular fracture in a war injury. **Conclusions**: The external fixator offers effective treatment for severe mandibular fractures in war-related injuries with low rates of complications and high success rates. Where a manufacturer external fixator is not available, orthopedic external fixators and self-crafted external fixators are used.

## 1. Introduction

At the end of 20th century, external fixation appliance seemed to be an unusual modality in the treatment of mandibular fractures and the treatment considered standard for mandible fractures was the rigid internal fixation [1,2,3].

There are situations where rigid internal situations are inappropriate, such as complex comminuted high-velocity gunshot fractures, where opening the surgical site and stripping off the periosteal in order to apply the plates and screws may devitalize bone, leading to sequestrum formation and infection [1]. In these situations, external fixation is suitable, and its benefits have been described by many authors [1,4,5,6]. This treatment has reduced morbidity when compared to the open treatment, allowing an adequate repair of bone and adjacent soft tissues and less potential for infection [7,8,9]. Bone plate stabilization of craniomaxillofacial injury caused by high-velocity missiles was attempted by surgeons in the Vietnam War, but the results seemed to be even worse than with wire fixation [3,8].

Other indications of external fixation are large bone defects waiting for secondary reconstruction caused by tumor resection, pathological mandibular fractures caused by infections, osteoradionecrosis, osteomyelitis and acute purulent infection [2,5,9,10,11,12,13]. External fixators can also be chosen as a second-choice treatment for cases of comminuted mandibular fractures initially treated with internal fixation, but which evolved with infection and the need to remove bone synthesis material [7,14,15,16]. Recently, the use of external fixators has been described for the treatment of condylar fractures [7,8,9,10,11,12,13,14,15,16,17,18,19].

Lambotte, at the beginning of the twentieth century, initially developed the external fixator for trauma management of the limbs [20]. Between 1934 and 1948, Ginestet transposed the technique to the maxillofacial skeleton [21,22].

This transition from orthopedic to maxillofacial applications was further advanced in the mid-20th century by pioneers such as Roger Anderson, who introduced a system of external fixation adapted to the craniofacial skeleton, particularly during the management of complex facial injuries in military settings. His design, later known as the Anderson device, formed the basis for further innovations in mandibular stabilization. Subsequently, Vladimir Rudko contributed to refining the technique and adapting it for routine use in civilian maxillofacial trauma, promoting minimally invasive principles and emphasizing functional recovery. These historical developments laid the foundation for the modern resurgence of external fixation in the management of mandibular war injuries [20].

Several types of mandibular external fixation devices exist. The oldest type is a modified Roger Anderson device, which is considered to be uniphasic and consists of two percutaneous pins on either side of the fracture linked together by a metal bar and connectors. The Joe Hall Morris device, used during the Korean War, consists of a biphasic system, where pins were connected with self-curing resin instead of rods, simplifying and lightening the fixator compared with models with rods [5]. Recently, newer versions of the uniphasic system were developed and have been made commercially available for use [9]. Unlike the traditional external fixator, those new versions of fixators are suitable for the mandible and adapting to the contours of this bone [13].

In the last decade, we have seen a steady increase in papers concerning the use of external fixators on the mandible because of the wars in Iraq, Afghanistan and other parts of the world where war is transpiring, high-velocity rifles are fired and missiles are used [5,7,23,24]. Although there has been a rebirth of interest in the use of the external fixator in maxillofacial surgery, as indicated by the plethora of case series published in the past 20 years, there is a lack of reliable information regarding the use of an external fixator on the mandible in war-related injuries. This systematic review aimed to summarize the external fixator used in mandibular war injury management, its benefits, side effects and complications. More specifically, this review focuses on the practical applications of external fixation in mandibular fractures caused by high-velocity ballistic trauma in military settings. It aims to synthesize available evidence regarding indications, technical feasibility, treatment outcomes and associated complications, in order to guide clinicians managing such injuries in austere or resource-limited environments.

## 2. Materials and Methods

### 2.1. Protocol

This systematic assessment was performed in accordance with the Preferred Reporting Items for Systematic Reviews and Meta-analysis (PRISMA) guidelines to assess the effectiveness of the external fixator in the treatment of war-related mandibular injury.

### 2.2. Search Strategies

To identify studies on external fixators used to treat war-related fractures of the mandible, a systematic search was conducted by two investigators in the databases of PubMed and Google Scholar from inception to December 2024. The search strategy used was defined with the PICO criteria: participants (Patients with war-related mandibular injury)—interventions (external fixation)—comparator (explosive device or high-velocity round projectile and initial treatment or definitive treatment or associated bone and soft tissue treatment)—outcome (time to bone healing, bone healing with return to normal function and facial morphology, non-union, infection of the pin insertion site, limitation of mouth opening). The search strategy used the following algorithm: ((“external fixators”[MeSH Terms] OR (“external”[All Fields] AND “fixators”[All Fields]) OR “external fixators”[All Fields] OR (“external”[All Fields] AND “fixator”[All Fields]) OR “external fixator”[All Fields]) AND (“human s”[All Fields] OR “humans”[MeSH Terms] OR “humans”[All Fields] OR “human”[All Fields]) AND (“mandible”[MeSH Terms] OR “mandible”[All Fields] OR “mandibles”[All Fields] OR “mandible s”[All Fields])) AND ((fha[Filter]) AND (humans[Filter]) AND (english[Filter])), utilizing Medical Subject Heading (MeSH) terms. A manual search was also performed on the references of the retrieved articles for additional relevant works, reviewing abstracts for potential full-text analysis and inclusion.

Due to the focus of this review on practical use in low-resource or conflict settings, the database selection was restricted to PubMed and Google Scholar, which are both freely accessible. This was a deliberate methodological choice to ensure reproducibility and feasibility of the review process under austere conditions. The potential impact of this limitation is acknowledged in the discussion.

### 2.3. Eligibility Criteria

The search was confined to English language literature reporting the use of the external fixator to treat military or war-related mandibular injuries on human subjects with no time restriction regarding publication date. The study encompassed all published prospective, retrospective, case series and case report studies. If publications combined different modalities of treatment for war-related mandibular fracture (open reduction and internal fixation, maxillomandibular fixation and external fixation), the data related to external fixation had to be isolated and included.

Laboratory or animal research, studies on cadavers and technical notes were excluded for this review. Studies using an external fixator to treat low-velocity mandibular gunshot injuries outside of war zone (peace time) or regarding the use of an external fixator to treat other traumatic mandibular fractures not obtained from gunshot were excluded. The use of an external fixator for isolated injuries of mandibular condyle was excluded. Studies regarding upper jaw or other facial bone fracture war-related injuries treated by external fixator were also excluded.

### 2.4. Study Selection

After conducting the search, duplicate entries were removed before the screening. The remaining articles were then screened for relevance based on their title and abstracts. The full texts of the shortlisted abstracts considered for full review were downloaded and reassessed for final inclusion, adhering to the predefined criteria. A second reviewer repeated the screening process to validate the results. Any discrepancies between the reviewers were reconciled.

### 2.5. Data Extraction and Analysis

The extracted data were the first author’s name, publication date, country of origin, the study design, the age and gender, the indication as initial or definitive fixation, patient count, the mechanism of war injury, fracture details including bone loss and soft tissue loss, the follow-up period, type of external fixator used, the fixation duration, the associated treatment for bone and soft tissue, the outcome and complications.

Should an article lack the necessary information, the relevant cells were labeled as ‘Not Available’ (NA). We undertook only the description and qualitative synthesis of the identified studies due to the absence of homogeneous randomized comparative studies.

### 2.6. Risk of Bias

The Newcastle–Ottawa scale (NOS) was used to assess the quality of the cohort studies included in this systematic review based on object selection, comparability and exposure. For case studies and case series studies included, we removed from the NOS the items that related to comparability and adjustment because those studies were non-comparative, and this resulted in 5 questions. We retained for the purpose of quality assessment the items that focused on selection, representativeness of cases and ascertainment of outcome and exposure. The number 1 or 0 was directly related to the quality of studies. For the cohort study, NOS has a total score of 9 and scores equal to or higher than 6 were considered to indicate high quality of the study. Lower scores corresponded to the lower quality of the article. For case studies and case series studies, we considered the report good or high methodological quality when all 5 criteria were fulfilled, moderate when 4 were fulfilled and poor or low methodological quality when ≤3 were fulfilled. This tool has previously been applied [25,26].

## 3. Results

### 3.1. Study Selections

**Figure 1:**
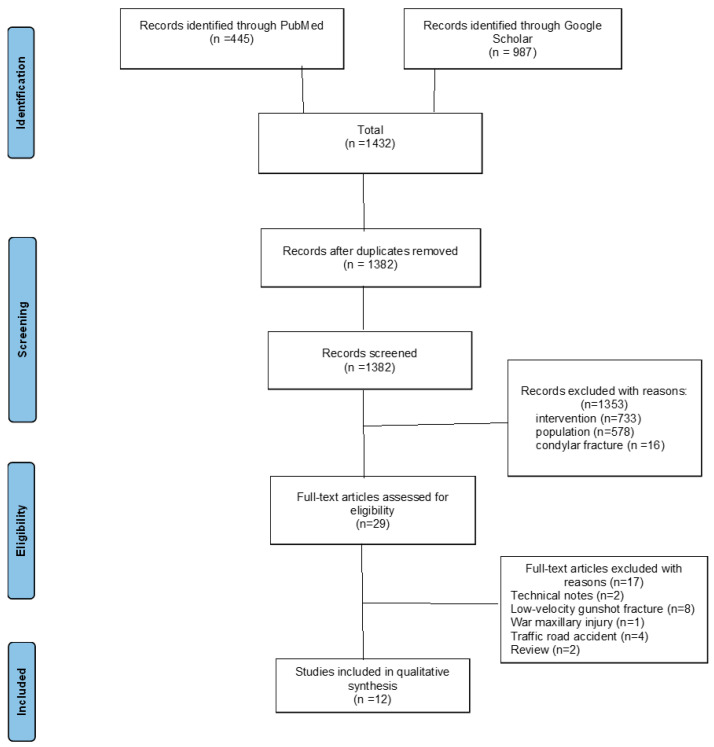
PRISMA flow-chart summarizes the search strategy used to make the final selection of publications for review.

A total of 1432 results were found in the search (Figure 1).

After removal of 50 duplicates, the titles of the remaining 1382 articles were screened independently by two researchers, after which 1353 were rejected due to irrelevancy. The full text of the remaining 29 articles were read in full and 17 publications were excluded. The reasons were because two were technical notes, two literature reviews, eight civilian gunshot injuries, one war maxillary injury, one motorcycle accident and three osteomyelitis complicated road accidents. In the end, 12 articles were selected for this systematic review (Table 1 and Table 2). The process of study selection and the reasons for exclusion are highlighted in a PRISMA flow-chart (Figure 1).

### 3.2. Study Characteristics

The 12 studies included in the qualitative synthesis were published from 1990 to 2023, including 10 papers between 2010 and 2023, with a marked increase over the last decade of the 12 studies. Four studies were retrospective cohort studies, four were case series studies, four were case studies. Six studies reported on injuries sustained in Iraq and Afghanistan published by UK-based groups operating in military hospitals, one study published by a Turkish group related to a case series of two Libyan civil war fighters transferred from the battlefield via airplane to Istanbul for trauma management and five studies reported on an unspecified combat zone. Moreover, eight studies concerned mandibular fractures treated exclusively using an external fixator and four studies combined an external fixator with other treatment methods.

The review included a total of 72 patients with mandibular war injuries who had undergone external fixation.

In this review, all the patients sustained injuries from an explosive device or high-velocity round and presented a heavily comminuted fracture of the mandible with large soft tissue defect and in three studies, significant loss of bone [2,13,14,23,27,28,29,30]. Only three studies reported improvised explosive device as the mechanism of injury [27,30,31]. In the other nine of twelve studies, gunshots with high-velocity round are reported to cause mandibular war injury treated by an external fixator. The external fixators were used during wartime either to definitively manage the mandibular war fracture in eight studies or as temporary treatment to initially stabilize the fracture. External fixation was an initial treatment in gunshot fractures of the mandible with large bone defect, which was followed by delayed open reduction internal fixation with osteochondral bone grafting and was performed in six studies [5,6,14,30,31,32]. The conversion time was mentioned by two authors; 2 months on the cases series presented by Elbir et al. in Turkey and 14 weeks on the case presented by Carvalho et al. in Brazil [14]. Table 1 and Table 2 summarize some the major characteristics of the papers included for review.

**Table 1:** Major characteristics of the papers included for review.

Authors (Year)	Study Design	Country	N. Patients	Type of External Fixator	Mechanism	Indication	Fracture Details	Outcome	Complications
Gibbons, 2011 [33]	Case report	UK	1	Custom II	High-velocity bullet	Definitive	Comminuted fracture	Bone healing, correct occlusion	None
Carvalho, 2019 [14]	Case series	NA	3	Orthopedic wrist	High-velocity bullet	2 Definitive, 1 Initial	Comminuted, 1 w/bone loss	Functional recovery, aesthetics restored	None
Zorman, 1990 [34]	Case series	NA	4	Orthopedic (Hoffman)	Missile injury	NA	NA	Good/excellent result	NA
Mc Millian, 2016 [30]	Case report	NA	1	NA	Explosive device	Definitive	Comminuted, large soft tissue defect	Excellent	None
Breeze, 2016 [28]	Case report	NA	1	NA	High-velocity bullet	Definitive	Comminuted, soft tissue maceration	Successful	Not reported
Elbir, 2023 [6]	Case series	Libya	2	Custom II	High-velocity bullet	Initial	Multifragmented	NA	None
Mc Veigh, 2010 [23]	Case series	UK	3	Custom II	Explosive device	Definitive	Comminuted, periosteal damage	Primary bone healing	NA
Marti-Flich, 2020 [5]	Retrospective cohort	France	24	Self-crafted	High-velocity bullet	5 Definitive, 19 Initial	NA	NA	Non-union (2 cases)
Venugopal 2011 [32]	Retrospective cohort	NA	10	NA	Explosive device	8 Definitive, 2 Initial	Comminuted	NA	NA
Maghalaes, 2020 [13]	Case report	NA	1	Orthopedic	High-velocity bullet	Definitive	Comminuted w/infection	Function and contour preserved	None
Breeze, 2011 [27]	Retrospective cohort	UK	22	NA	Explosive device	Definitive	NA	NA	NA
Ellis, 2003 [2]	Retrospective cohort	NA	17	NA	High-velocity bullet	NA	Comminuted	NA	Non-union (4 cases 23.5%)

**Table 2:** Major characteristics of the papers included for review (continuation and end).

Authors (Year)	Follow-Up/Duration of External Fixator (Week)	Associated Bone Treatment	Associated Soft Tissue Treatment
Gibbon, 2011 [33]	7 weeks	NA	NA
Carvalho 2019 [14]	14 weeks	Osteochondral bone graft and reconstruction with plate and screw: 1 patient	
Zorman, 1990 [34]	NA	NA	NA
McMillian, 2016 [30]	NA	NA	NA
Breeze, 2019 [28]	NA	Iliac bone graft	NA
Elbir, 2023 [6]	8 weeks (2 months)	Iliac bone graft and reconstruction plate	NA
Mc Veigh, 2010 [23]	NA	Miniplate osteosynthesis of substantial dentoalveolar fragments	NA
Marti-Flich, 2020 [5]	NA	NA	NA
Venugopal, 2011 [32]	24 months of follow-up	NA	NA
Maghalaes, 2020 [13]	NA	no	Intraoral and extraoral wound healed in second intention
Breeze, 2011 [27]	NA	NA	NA
Ellis, 2003 [2]	NA	NA	NA

### 3.3. External Fixator Device Available

Seven of the twelve studies mentioned the type of external device used [5,6,13,14,23,33,34]: three orthopedic external fixators (*n* = 3) [13,14,34], external fixators manufactured for the mandible (*n* = 3) [6,23,33], a self-crafted mandibular external fixator with components of at least three pins in each pone fragment connected with a breathing tube filled with self-cured resin which will rigidify the device (*n* = 1) [5].

### 3.4. Outcome and Fracture Union Duration

Overall, the external fixation device allowed the return to normal function and morphological aspect of the mandible [5,6,14,23,28]. Only three studies provided information about time duration of immobilization, which allows the external fixator to achieve bone healing ranging from 7 weeks [33] to 12 weeks [13,14]. Although reported in only a minority of studies, the average duration of external fixation in these high-velocity injuries ranged between 7 and 14 weeks, with longer durations generally associated with bone loss or staged reconstruction protocols.

### 3.5. Complications

Only three authors mentioned post-operative complications, whereas other authors did not experience any complications when they used external fixation to treat mandibular war injuries. Two authors reported non-union requiring bone graft reconstruction [2,5]. Venugopal et al. reported minor scarring and skin contractions after removal of the external fixation device was reported [32]. Three studies focused their attention to intraoperative technical problems during external fixation technique so as to avoid fracture of tooth roots and inferior alveolar nerve by positioning the pins closest to the base of the mandible [5,8,14].

#### Quality Assessment of Studies

The assessment of the methodological quality of the included case studies and case series studies is shown in Table 3. Four studies had good quality, two moderate quality and one poor quality. When assessing the quality of the cohort studies, the total score of all included studies, except for one, was greater than or equal to 6, indicating high-quality studies (Table 4).

## 4. Discussion

Multiple treatment options such as ORIF, MMF and external fixation are available for the treatment of mandible fractures. Our objective in conducting this systematic review was not only to catalog the use of external fixators in war-related mandibular trauma but also to clarify their specific role in clinical decision-making, whether as definitive or temporary stabilization—particularly in the context of high-velocity, comminuted fractures that pose a challenge for conventional osteosynthesis. At the end of 20th century, external fixation appliance seemed to be an unusual modality in the treatment of mandibular fractures. Contemporary military conflicts in Iraq and Afghanistan have once again demonstrated the usefulness of external fixation in the management of war mandibular injuries [27,33]. Gibbons, Breeze and McMillian, oral and maxillofacial surgeons from the UK, reported several case series and case report of patients with comminuted mandibular fracture war-related injuries sustained in Iraq and Afghanistan managed by external fixation [7,27,30,33,35,36,37,38,39,40,41,42].

We attempted to find answers to the following questions: What are the external fixators available for mandible fracture management? What are the indications and fracture patterns for using external fixation in a mandibular war injury? What are the benefits and reported outcomes of external fixators reported in the literature? What are complications associated with external fixation in war mandibular injuries?

In search of the answer to the first question, we found that there are three main external fixators available for mandibular war fractures: the manufacturer external fixator, the self-crafted Joe Morris biphasic fixator and the orthopedic external fixator. Orthopedic fixators for hand and wrist fractures were successfully used in the treatment of mandibular injuries [14]. The advantages of orthopedic external fixators are that the kit is usually available, and they are versatile, expedient and simple to apply, although they are bulky to wear and the bar shapes and pins are not customized for the mandible [13,43,44,45]. Modern incarnations of external fixators of the mandible (manufacturer mandibular external fixators), such as those produced by Synthes©, have titanium bars that are shaped to conform to the contours of the mandible, are operator-friendly and are associated with improved patient comfort and tolerance, although they are expensive [5,23]. The system is adjustable and lightweight [11,13,33,46] and it is well suited for use in the modern combat surgery environment. The self-crafted Joe Hall Morris biphasic appliance, described Korean War, consists of a biphasic system where pins are connected with self-curing resin instead of rods. Its inconvenience is that it is not quickly applied and once in place, it cannot be adjusted [5,33,47,48].

In terms of practical application, several technical considerations must be kept in mind when using external fixation in mandibular trauma. Pin placement should avoid vital anatomical structures such as tooth roots, mental foramen and the inferior alveolar nerve. Most authors recommend placing pins close to the inferior border of the mandible, in the basal bone, where cortical thickness allows for better purchase and lower risk of iatrogenic injury. Bicortical pin placement is preferable for stability [14]. In austere or military settings, orthopedic fixators or improvised constructs (such as breathing tubes filled with resin) can be effectively adapted for mandibular use, although they may lack the conformity and comfort of modern mandibular-specific devices [5]. Despite their limitations, these adaptations remain valuable where commercial devices are unavailable. Attention must also be paid to soft tissue clearance and the need for pin site hygiene to reduce the risk of secondary infection [32].

In regard to our second question about indications and fracture patterns for using external fixation, we found that all patients reported presented complex comminuted fractures with soft tissue damage from high-velocity missiles and explosive devices [24]. These complex comminuted fractures are associated with large periosteal, muscle or mucosal damage. The placement of foreign bodies such as plates and screws in this setting requires wide periosteal stripping at the fracture site that may compromise the blood supply and increase the infection risk. The use of external fixation provides an optimal environment allowed to obtain complete bone healing [3,7,8,10,23,31].

For the third question regarding the benefits and reported outcomes, it has been shown that external fixation yields satisfactory outcomes even without transitioning to internal osteosynthesis. External fixation is recommended as a definitive treatment option, as it eliminates the necessity for a second surgical procedure, thereby indirectly reducing patient burdens associated with awaiting surgery and avoiding the risks linked to subsequent operations and anesthesia in low-income countries [30,33]. External fixation is used for temporary fixation of highly comminuted mandibular fractures with bone defects when bone grafting is required. The initially adequate stabilization of facial fractures is necessary to prevent collapse and fibrosis that are very difficult to treat once established [14,23].

When comparing external fixation to other commonly used techniques for mandibular fracture management, particularly in war-related injuries, several advantages become apparent. Open reduction and internal fixation (ORIF) provide rigid stabilization and can restore anatomy and occlusion precisely, but they require extensive surgical exposure, periosteal stripping and are contraindicated in contaminated or infected fields. In contrast, external fixation offers stabilization without compromising the soft tissue envelope, which is essential in high-velocity comminuted injuries with severe soft tissue loss or when infection is present. Maxillomandibular fixation (MMF), while less invasive, does not provide direct fracture stabilization and can be problematic in polytraumatized or intubated patients. In military or austere environments, where delayed reconstruction is often necessary, external fixation serves as an ideal temporizing or even definitive solution. It reduces operative time, allows soft tissue healing before reconstruction, and facilitates oral hygiene and feeding when compared to prolonged MMF. The choice of technique should be guided by injury severity, contamination and logistical constraints [2,7,23,27,31].

The duration of external fixation in war-related mandibular injuries is influenced by the complexity of the fracture, the presence of bone loss and the timing of definitive reconstruction. While only a few of the studies included in our review reported fixation times, available data suggest a typical range from 7 to 14 weeks. These findings are consistent with a large retrospective series by Rose et al., which demonstrated that fixation duration depends on the mechanism of injury [49]. In their study, gunshot wounds had an average external fixation duration of 79 days, compared to 107 days in cases of failed ORIF with non-union and osteomyelitis, and shorter durations for motor vehicle accidents (18 days) and pathologic fractures (16 days). This supports the idea that high-velocity ballistic trauma, as seen in war injuries, often requires prolonged stabilization periods due to the extent of comminution, bone loss and infection risk [49].

Although the included studies rarely detailed the rehabilitation process or long-term functional follow-up, it is generally accepted that the management of high-velocity mandibular injuries treated with external fixation involves a phased approach. Based on clinical reasoning and available case reports, rehabilitation can be conceptually divided into three stages: (i) initial stabilization, during which external fixation provides mechanical stability and allows for wound management; (ii) the intermediate phase, focusing on soft tissue healing, infection control and monitoring of bone consolidation; and (iii) the delayed or definitive reconstruction phase, which may include bone grafting, dental rehabilitation and physiotherapy aimed at restoring occlusion, mandibular function and aesthetics. While our review confirms that most patients achieved satisfactory anatomical and functional outcomes, further research is needed to define the optimal timing and protocols for rehabilitation.

Regarding the last question we attempted to answer, the complications associated with external fixation in this review were non-union requiring bone graft reconstruction [2,5], minor scarring and skin contractions after removal of external fixation device [32]. A study shows that external fixation had an extremely high complication rate in the management of high-velocity injury. This may be attributed to the complexity of the injury itself and not necessarily the use of the external fixation device [50].

Although our systematic review did not identify studies reporting histological findings of bone stumps or regenerated tissue following high-velocity mandibular gunshot wounds, the available orthopedic and military trauma literature describes typical features of bone exposed to ballistic trauma. These include areas of necrosis, microfractures, thermal and mechanical devitalization at the wound margins, and extensive periosteal stripping, which compromise the biological environment for healing. These devitalized bone fragments often require thorough debridement to prevent chronic infection or non-union. Furthermore, the regenerated bone that forms under external fixation in such settings may show delayed remodeling and irregular architecture, particularly when associated with infection or insufficient stabilization. These observations support a staged approach to reconstruction and the value of external fixation in allowing the bone and soft tissues to recover prior to definitive management.

### Strength and Study Limitations

This is the first systematic review to describe the use of external fixators for the treatment of mandibular war fractures. This review demonstrates the advantages of external fixation in this specific group of patients, but it also faces several limitations. Firstly, as most articles included in this study studies were case series, the evidence level is considered low. Secondly, as most of the operations were accomplished on an emergency basis and war context, it is difficult to conduct randomized trials. However, more comparative and prospective studies can be conducted in the future to validate the outcome shown. Another limitation is the database selection. The exclusion of proprietary databases such as Scopus or Embase may have led to the omission of some relevant studies. However, this was an intentional choice to prioritize open-access and replicability in the context of low-resource or war-affected settings, which aligns with the practical aim of the review.

## 5. Conclusions

This review establishes the current role of external fixation in maxillofacial fracture treatment. The use of external fixation for the treatment of mandibular fractures is recommended and appropriate to treat severe mandibular fractures with high success rate in military practice. In addition, it provides adequate fracture stabilization for temporary or staged treatment and can be converted to more stable constructs for definitive management. This review reports lower complication rates following external fixation even in severe mandibular fractures. Where the manufacturer external fixator is not available, orthopedic external fixators and self-crafted external fixators are used.

## Figures and Tables

**Table 3:** Methodological quality of included cases studies and cases series studies.

Reference	Question 1		Question 2		Question 3		Question 4		Question 5		Overall Quality
Gibbon, 2011 Case report [33]	☺		☺		☺		☺		☺		Good
Carvalho 2019, case series [14]	☺		☺		☺		☺		☺		Good
Zorman 1990, case series [34]	☺		☺		☺		☺		☺		Low
McMillian 2016, case report [30]	☺		☺		☺		☺		☺		Low
Breeze 2016, case report [28]	☺		☺		☺		☺		☺		Moderate
Elbir 2023, case series [6]	☺		☺		☺		☺		☺		Good
Mc Veigh 2010, case series [23]	☺		☺		☺		☺		☺		Good
Maghalaes 2020, case report [13]	☺		☺		☺		☺		☺		Moderate

Questions addressed for assessment of methodological quality: 1. Did the patient(s) represent the whole case(s) of the medical center? 2. Was the diagnosis correctly made? 3. Was the other important diagnosis excluded? 4. Were all the important data cited in the report? 5. Was the outcome correctly ascertained?

**Table 4:** Methodological quality of included cohort studies.

Reference	Selection				Comparability	Outcome			
	Representativeness of the Cases	Ascertainment of Exposure	Selection of Non-Exposed	Outcome Was Not Present at the Start of Study	Comparability of Cohorts	Assessment of Outcome	Sufficient Follow-Up Time	Adequacy of Follow-Up of Cohorts	Overall Quality
Marti-Flich 2020, retrospective cohort study [5]	0	0	1	1	1	1	1	1	5
Venugopal 2011, retrospective cohort study [32]	1	1	0	1	2	1	1	1	8
Breeze 2011, Retrospective cohort study [27]	1	1	1	1	2	1	1	1	9
Ellis 2003, retrospective cohort study [2]	1	1	1	1	2	1	1	1	9

## Abbreviations

The following abbreviations are used in this manuscript:

IMF	Intermaxillary fixation
ORIF	Open reduction and internal fixation
MMF	Maxillomandibular fixation
NA	Not available
UK	United Kingdom
NOS	Newcastle–Ottawa Scale
EF	External fixation

## References

[1] Kazi A.A., Lee T.S., Vincent A., Sokoya M., Sheen D., Ducic Y. (2019). The role of external fixation in trauma and reconstruction of the mandible in the age of rigid fixation. Facial Plast. Surg..

[2] Ellis E., Muniz O., Anand K. (2003). Treatment considerations for comminuted mandibular fractures. J. Oral Maxillofac. Surg..

[3] Chrcanovic B.R. (2013). Open versus closed reduction: Comminuted mandibular fractures. Oral Maxillofac. Surg..

[4] Fleming I.D., Morris J.H. (1969). Use of acrylic external splint after mandibular resection. Am. J. Surg..

[5] Marti-Flich L., Schlund M., Raoul G., Maes J.-M., Ferri J., Wojcik T., Nicot R. (2020). Twenty-four years of experience in management of complex mandibular fractures with low cost, custom-made mandibular external fixation: A 65-patient series. J. Stomatol. Oral Maxillofac. Surg..

[6] Elbir B., Kolsuz N., Varol A. (2023). External mandibular fixation for gunshot fractures: Report of 2 cases. Ulus. Travma. Acil. Cerrahi. Derg..

[7] Torres L.H.S., Uchoa C.P., Cavalcante M.B., Jardim V.B.F., Rodrigues É.D.R., Pereira R.V.S., Côvre L.M., do Egito Vasconcelos B.C., e Silva E.D.D.O., Pereira Filho V.A. (2020). Colles external fixator as alternative in comminuted mandibular fractures treatment. Res. Soc. Dev..

[8] Cornelius C.-P., Augustin J.B., Sailer L.-K. (2009). External pin fixation for stabilization of the mandible—Comeback of a method: Historical review and first experiences with the ‘mandible external fixator’. Oral Maxillofac. Surg..

[9] Braidy H.F., Ziccardi V.B. (2009). External fixation for mandible fractures. Atlas Oral Maxillofac. Surg. Clin..

[10] Holmes S., Hardee P., Anand P. (2002). Use of an orthopaedic fixator for external fixation of the mandible. Br. J. Oral Maxillofac. Surg..

[11] de Alencar M.G.M., De Bortoli M.M., da Silva T.C.G., e Silva E.D.d.O., Laureano Filho J.R. (2018). Suitability of wrist external fixator for treatment of mandibular fracture. J. Craniofacial Surg..

[12] Alpert B., Tiwana P.S., Kushner G.M. (2009). Management of comminuted fractures of the mandible. Oral Maxillofac. Surg. Clin. N. Am..

[13] Magalhães G.P., de Carvalho M.L., Ingryd J., de Sousa T., dos Santos J.Z.L.V. (2020). Mandibula Fracture by Fire Weapon Projectile: Surgical Treatment Through the Use of External Fixers: Case Report. IOSR J. Dent. Med. Sci. (IOSR-JDMS).

[14] Carvalho P.H.R., da Hora Sales P.H., da Rocha S.S., Cavalcanti A.M.M., de Jesus Rodrigues Mello M., Junior J.M.S.M. (2019). Treatment of comminutive fractures by firearm projectiles with adapted wrist external fixator. Oral Maxillofac. Surg..

[15] Barreda Hale M., Romero-Araya P., Cea Herrera M., Espinoza D., Castro N., Castro J., Serandour G. (2021). Computer-assisted planning with 3D printing for mandibular reconstruction caused by a mandibular fracture with secondary osteomyelitis: A Case Report. Clin. Case Rep..

[16] Mahdian N., Onderková A., Brizman E., Pavlíková G., Vlachopulos V., Drahoš M., Foltán R. (2020). External fixation greatly improves outcomes in the surgical treatment of osteoradionecrosis of the jaws without affecting quality of life: A five-year retrospective study. Br. J. Oral Maxillofac. Surg..

[17] Cascone P., Spallaccia F., Arangio P., Vellone V., Gualtieri M. (2017). A modified external fixator system in treatment of mandibular condylar fractures. J. Craniofacial Surg..

[18] Iannetti G., Cascone P. (1995). Use of rigid external fixation in fractures of the mandibular condyle. Oral Surg. Oral Med. Oral Pathol. Oral Radiol. Endod..

[19] Cascone P., Marcozzi M.M., Ramieri V., Bosco G., Vellone V., Spallaccia F. (2017). Mandibular Condylar Fractures in Children: Morphofunctional Results After Treatment with External Fixation. J. Craniofac. Surg..

[20] Hernigou P. (2017). History of external fixation for treatment of fractures. Int. Orthop..

[21] Mohamed A., Mepani V., Sharma V. (2020). Use of an endotracheal tube in the biphasic fixation of a mandibular fracture. Br. J. Oral Maxillofac. Surg..

[22] Mukerji R., Mukerji G., McGurk M. (2006). Mandibular fractures: Historical perspective. Br. J. Oral Maxillofac. Surg..

[23] McVeigh K., Breeze J., Jeynes P., Martin T., Parmar S., Monaghan A.M. (2010). Clinical strategies in the management of complex maxillofacial injuries sustained by British military personnel. BMJ Mil. Health.

[24] Wilkening M.W., Patel P.A., Gordon C.B. (2012). External fixation in a low-velocity gunshot wound to the mandible. J. Craniofacial Surg..

[25] Bazerbachi F., Haffar S., Hussain M.T., Vargas E.J., Watt K.D., Murad M.H., Chari S., Dayyeh B.K.A. (2018). Systematic review of acute pancreatitis associated with interferon-α or pegylated interferon-α: Possible or definitive causation?. Pancreatology.

[26] Haffar S., Bazerbachi F., Prokop L., Watt K.D., Murad M.H., Chari S.T. (2017). Frequency and prognosis of acute pancreatitis associated with fulminant or non-fulminant acute hepatitis A: A systematic review. Pancreatology.

[27] Breeze J., Gibbons A.J., Hunt N.C., Monaghan A.M., Gibb I., Hepper A., Midwinter M. (2011). Mandibular fractures in British military personnel secondary to blast trauma sustained in Iraq and Afghanistan. Br. J. Oral. Maxillofac. Surg..

[28] Breeze J., Parmar S., Monaghan A., Idle M.R., Monaghan A.M. (2016). 71 High-energy ballistic injuries to the face. Challenging Concepts in Oral and Maxillofacial Surgery: Cases with Expert Commentary.

[29] Tonn C.R., Ward M.L., Abadie W.M., Lally J.W., Bevans S.E., Henry L.R. (2020). Military Injuries to the Head and Neck—Implications for Practice in Resource Constrained Environments. Oper. Tech. Otolaryngol. Head Neck Surg..

[30] McMillan K., Martin T., Idle M.R., Monaghan A.M. (2016). 77 Reconstructive challenges following blast injuries to the facial soft tissue and skeleton. Challenging Concepts in Oral and Maxillofacial Surgery: Cases with Expert Commentary.

[31] Tucker D.I., Zachar M.R., Chan R.K., Hale R.G. (2013). Characterization and management of mandibular fractures: Lessons learned from Iraq and Afghanistan. Atlas Oral Maxillofac. Surg. Clin. N. Am..

[32] Venugopal A., Bhatt V., Williams R., Sharp I., Parmar S., Monaghan A. (2011). The use of external fixators and intermaxillary fixation in comminuted fractures of the facial skeleton. Br. J. Oral Maxillofac. Surg..

[33] Gibbons A.J., Mackenzie N., Breederveld R.S. (2011). Use of a custom designed external fixator system to treat ballistic injuries to the mandible. Int. J. Oral Maxillofac. Surg..

[34] Zorman D., Godart P.A., Kovacs B., Andrianne Y., Daelemans P., Burny F. (1990). Treatment of mandibular fractures by external fixation. Oral Surg. Oral Med. Oral Pathol..

[35] Gibbons A.J., Mackenzie N. (2010). Lessons learned in oral and maxillofacial surgery from British military deployments in Afghanistan. J. R. Army. Med. Corps..

[36] Breeze J., Gibbons A.J., Opie N.J., Monaghan A. (2010). Maxillofacial injuries in military personnel treated at the Royal Centre for Defence Medicine June 2001 to December 2007. Br. J. Oral Maxillofac. Surg..

[37] Gibbons A.J., Breeze A.F. (2011). The face of war: The initial management of modern battlefield ballistic facial injuries. J. Mil. Veterans’ Health.

[38] Breeze J., Monaghan A.M., Williams M.D., Clark R.N.W., Gibbons A.J. (2010). Five months of surgery in the multinational field hospital in Afghanistan with an emphasis on oral and maxillofacial injuries. J. R. Army Med. Corps..

[39] Breeze J., Gibbons A.J., Combes J.G., Monaghan A.M. (2011). Oral and maxillofacial surgical contribution to 21 months of operating theatre activity in Kandahar Field Hospital: 1 February 2007–31 October 2008. Br. J. Oral. Maxillofac. Surg..

[40] Breeze J., Tong D., Gibbons A. (2017). Contemporary management of maxillofacial ballistic trauma. Br. J. Oral Maxillofac. Surg..

[41] Breeze J., Blanch R., Baden J., Monaghan A.M., Evriviades D., Harrisson S.E., Roberts S., Gibson A., MacKenzie N., Baxter D. (2018). Skill sets required for the management of military head, face and neck trauma: A multidisciplinary consensus statement. J. R. Army Med. Corps..

[42] Peleg K., Aharonson-Daniel L., Stein M., Michaelson M., Kluger Y., Simon D., Noji E.K., Israeli Trauma Group (2004). Gunshot and explosion injuries: Characteristics, outcomes, and implications for care of terror-related injuries in Israel. Ann. Surg..

[43] Deininger C., Hofmann V., Necchi M., Deininger S., Wichlas F. (2022). Off-Label Treatment for Severe Craniomaxillofacial Fractures in Low-Income Countries—A Novel Operation Method with the External Face Fixator. J. Clin. Med..

[44] Breeze J., Bryant D. (2009). Current concepts in the epidemiology and management of battlefield head, face and neck trauma. BMJ Mil. Health.

[45] Will M.J., Goksel T., Stone C.G., Doherty M.J. (2005). Oral and maxillofacial injuries experienced in support of Operation Iraqi Freedom I and II. Oral Maxillofac. Surg. Clin. N. Am..

[46] Neupert E.A., Boyd S.B. (1991). Retrospective analysis of low-velocity gunshot wounds to the mandible. Oral Surg. Oral Med. Oral Pathol..

[47] Louis P.J., Fernandes R. (2001). Temporary stabilization of the mandible with an external fixation device. J. Oral Maxillofac. Surg..

[48] Shuker S.T. (2013). Interrami intraoral fixation technique for severe mandibular rifle fragmented bullet injury management. J. Craniofac. Surg..

[49] Rose M.J., Shanti R.M., Iocca O., Rasa M., Ziccardi V.B. (2025). Retrospective analysis of external pin fixation of mandibular fractures: A 25-year single institution experience. J. Craniomaxillofac. Surg..

[50] Rhodes J., Lew F., Agarwal V., Cho B. (2017). Gunshot Wounds to the Mandible: A 21-Year Urban Trauma Center’s Experience. Plast. Surg. Mod. Tech..

